# Procedures for systematic capture and management of analytical data in academia

**DOI:** 10.1016/j.acax.2019.100007

**Published:** 2019-02-15

**Authors:** Jan Potthoff, Pierre Tremouilhac, Patrick Hodapp, Bernhard Neumair, Stefan Bräse, Nicole Jung

**Affiliations:** a Steinbuch Centre for Computing, Karlsruhe Institute of Technology, Hermann-von-Helmholtz-Platz 1, 76344, Eggenstein-Leopoldshafen, Germany; b Institute of Toxicology and Genetics, Karlsruhe Institute of Technology, Hermann-von-Helmholtz-Platz 1, 76344, Eggenstein-Leopoldshafen, Germany; c Institute of Organic Chemistry, Karlsruhe Institute of Technology, Fritz-Haber-Weg 6, 76131, Karlsruhe, Germany

**Keywords:** Research data management, Information and management systems, Analytical data, Infrastructure, ELN

## Abstract

Data management in universities is a challenging endeavor in particular due to the diverse infrastructure of devices and software in combination with limited budget. Nevertheless, in particular the analytical measurements and data sets need to be stored if possible digitally and in a well-organized manner. This manuscript describes how scientists can achieve a data management workflow focusing on data capture and storage by small adaptions to commonly used systems. The presented method includes data transfer options from ubiquitous devices like NMR instruments, GC (MS) or LC (MS), IR and Raman, or mass spectrometers to a central server and the visualization of the available data files in an electronic lab notebook (ELN). The given instruments were chosen according to the needs of synthetic chemists, in particular devices needed in organic, inorganic and polymer chemistry where single data files in the range of several megabytes per data set are produced. Altogether, three different data transfer systems were elaborated to allow a flexible handling of different devices running with different proprietary software: The first procedure allows data capture via the use of a mail server as data exchange point. With the second procedure, data are automatically mirrored from a local file folder to a central storage server where new files are monitored and processed. The third procedure was designed to transfer data with manual support to a central server which is supervised to register new information. All components that are necessary to install and use the herein elaborated functions are available as Open Source and the designed workflows are described step by step to facilitate the adaption of procedures in other universities accordingly if desired.

## Highlights

• Analytical data management is of high importance to support scientific work.
• New procedures in academia are necessary to meet current requirements.
• Data capture and storage with focus on synthetic (in)organic and polymer chemistry.
• Implementation of data transfer options from devices to a central server.
• Visualization of the available data files in an electronic lab notebook (ELN).

## Graphical abstract

**Figure:**
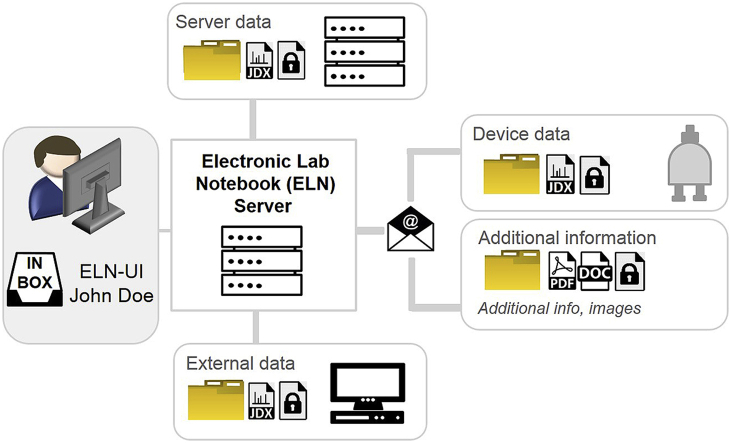

## 1 Introduction

Systematic data collection and management is of high importance in research institutions, in particular due to the increasing requirements to develop and meet research data management plans. Systems that support researchers in this aim contribute to the improvement of the overall data structure and help scientists to keep the overview of analytical files. Additionally, management systems for analytical data allow the systematic storage of digital data which is of high importance e.g. for the accessibility of research data in compliance with the FAIR data [1] principles. In contrast to this need, the systematic capture and storage of analytical data in natural sciences and in experimental or analytical laboratories can be a challenging endeavor even for non-big data research due to the manifold sources of data and the use of different devices for their acquisition. Therefore, larger companies usually invest in Laboratory and Information Management Systems (LIMS) which allow to control the data workflows, data tracking, and data transfer. Some of the well-known systems are STARLIMS (Abbott Informatics Corporation) [2], Limsophy (AAC Infotray) [3], Biovia LIMS (Accelrys) [4], SampleManager (Thermo Fisher Scientific) [5], webLIMS (LabLynx) [6] or LabsoftLIMS (Computing Solutions) [7]. A LIMS allows to reduce the errors that are inevitably associated with manual data handling. It prevents the loss of data, and improves the work efficiency, in particular with a high throughput of data. The disadvantages of a LIMS are in general the high costs of user licenses and the software itself as well as adaptions that are in most of the cases necessary. In addition, there is a need to train users at a high technical level to troubleshoot issues if one wants to avoid a permanent consulting of the software supplier. More recently, Open Source LIMS solutions like simpleLIMS [8], open-LIMS [9] or bika-LIMS [10] were developed. These Open Source LIMS are in particular attractive for smaller companies (SMEs) and academic institutions as their installation and maintenance are free of licensing or external service costs. As data management includes not only data from devices or any digital sources, many LIMS are combined with an electronic lab notebook (ELN) that allows the collection and management of written information, values and images. Examples are labcollector (Agile Bio) [11], Labware LIMS-ELN [12] or openBIS [13]. The combination of a LIMS with an ELN offers important advantages especially in an academic environment where non-standardized workflows have to be recorded and additional data from external sources have to be captured. However, even when considering the benefits of LIMS and ELN, and the combination of both, only very few universities run either commercial (see above), free, or Open Source management systems. The reasons for this lack of managing systems, even more pronounced for LIMS, might be found in the diverse infrastructure and institution boundaries of the universities, the related budget issues and/or long-term maintenance responsibilities. Another reason that explains the rare use of Open Source solutions might be the age of the equipment and the challenges resulting in this: Academic groups very often use devices from diverse vendors. Though the main hardware lifetime can be relatively long, software obsolescence often hinders a prolonged use: the equipment is controlled by ageing software. Sometimes it can be upgraded at additional cost but in the worst case, both the hardware and the software need to be renewed to allow their integration into the workflows of a modern LIMS. The integration of workflows to a LIMS requires not only the acquisition of new infrastructures but also the adaptation of existing procedures. The efforts to implement these changes is a reason why academic groups do not benefit from currently available methods for well-organized data capture and management. In the herein presented solution, we describe a very simple procedure for the management of data in universities. We describe a workflow that has been established at the Karlsruhe Institute of Technology (KIT) to meet the requirements of a chemistry research group using analytical devices that are common in the field of synthetic, organic, inorganic and polymer chemistry. We aimed for a simple but beneficial contribution to data management that can be adopted with ease by other research chemistry groups or universities. Therefore, special importance was given to the general applicability of the procedures, their effortless implementation and the clear directive to run a working infrastructure. The instruments that were implemented exemplarily were chosen to show the principles of the overall concept that can be applied for additional other instruments. The presented solution was integrated into the Chemotion ELN, which provides organic chemists with a user interface to manage their data.

## 2 Results and discussion

**Fig. 1:**
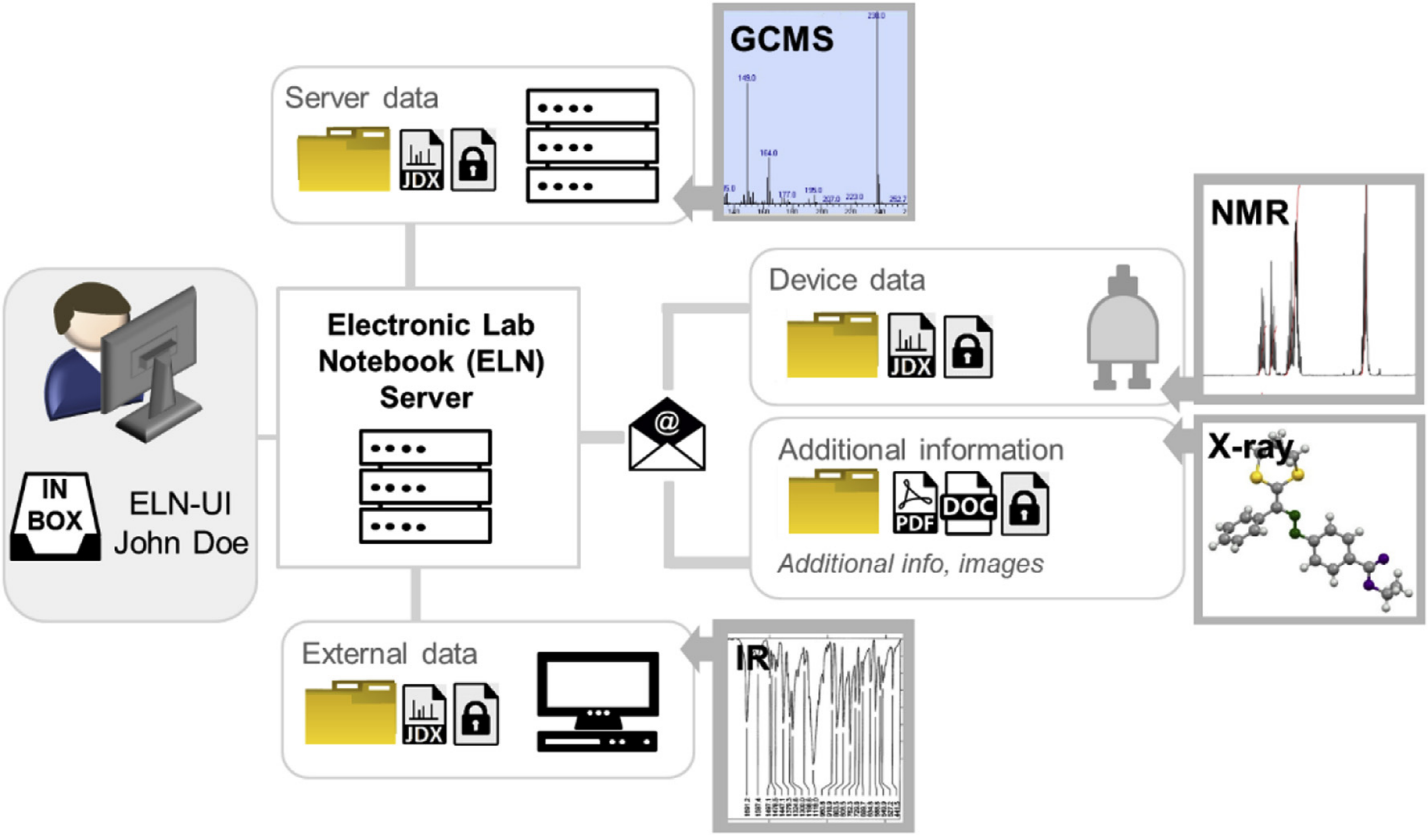
Overview of the three procedures to integrate research data from (analytical) devices to an ELN. The given analytical methods are chosen as examples to illustrate the procedure. They can be exchanged depending on the local infrastructure.

In chemistry, information to be collected consists of the calculations and observations of the researchers and data from mainly analytical devices. The data generated by devices is often recorded in a different way (concerning file types, acquisition procedures) and access to the devices is often restricted. Typical instruments that are used in especially synthetic chemistry include NMR spectrometers, GC (gas chromatography) instruments with and without mass coupling, HPLC (high performance liquid chromatography) instruments with and without mass coupling, elemental analyzers, IR (infrared) spectrometers, and UV-VIS (ultraviolet–visible) spectrometers. The data is recorded either by the scientist as the direct device operator or by another person providing the measurement as a service. The data is often stored on local hardware that is connected to a local network. Other instruments store data locally but are not connected to any network or server because of a lacking infrastructure, obsolete hardware, and/or security concerns. This causes additional problems for the flow of data and the availability of analytical datasets. A suitable infrastructure for data management should therefore consist of a data management system like an ELN providing a user interface (UI) to plan research and collect all information (independent of its origin) and a backend architecture that allows the transfer of all relevant data to the ELN. The use of an ELN as user interface for the management of analytical research data coming from different devices is particularly beneficial for organic chemistry, as data can be assigned to chemical structures and their preparation. Several electronic lab notebooks (ELNs) have been developed during the last years offering intelligent solutions for the documentation of chemistry research [[14], [15], [16]]. Examples of systems in chemistry that offer the necessary support of chemical structures are the PerkinElmer E-Notebook for Chemistry [17], Indigo-ELN [18], LabTrove [[19], [20], [21]], and OpenEnventory [22]. Our group developed its own ELN (Chemotion-ELN) which is running in several labs of the Karlsruhe Institute of Technology (KIT) [23]. We therefore decided to use the Chemotion ELN as proof of concept for the herein designed backend architecture. Referring to the diverse origin of data in chemistry labs, we implemented three different procedures to allow the availability of data to the ELN server (Fig. 1). The first procedure includes the transfer of data (preferably small data) by a mailing system which manages the capture of NMR data and additional information that is sent by mail to the email account of the ELN. A second procedure was designed for the transfer of data from a local server or computer with network connection to the ELN server. The last procedure was implemented for the transfer of data from devices without network access (due to safety standards, location issues or software-related issues). For the transfer of data from devices without network connectivity, we implemented a procedure which requires the manual collection of data (for example with a portable data storage e.g. an USB flash drive) and its transfer to the ELN via WinSCP running on a computer with network connection. These three procedures have been implemented for several devices and have been tested with many data formats. It was shown that the combination of the three procedures is suitable to improve the situation in synthetic chemistry labs in academia with respect to data transfer options. The overall method was designed to allow a flexible addition of diverse instruments. Devices that have not been used in this study can be included to the workflow described in one of the three procedures if some general prerequisites are fulfilled (please see details in the following description of the procedures).

### 2.1 Execution of the data collection: task queueing and frequency

The data collection tasks are all registered as ‘delayed_job’ tasks. The ‘delayed_job’ gem is a ruby package allowing queuing and executing background tasks asynchronously. The task execution is then independent from the ELN web server activities though each task is persisted in the ELN DB up to its execution. The data collection configuration accepts a cron schedule typed parameters that configures the task execution frequency. After a task is successfully executed it is requeued for a later time according to the schedule configuration. Each data collection procedure uses its own schedule parameter.

### 2.2 Mailcollector (Procedure 1)

**Fig. 2:**
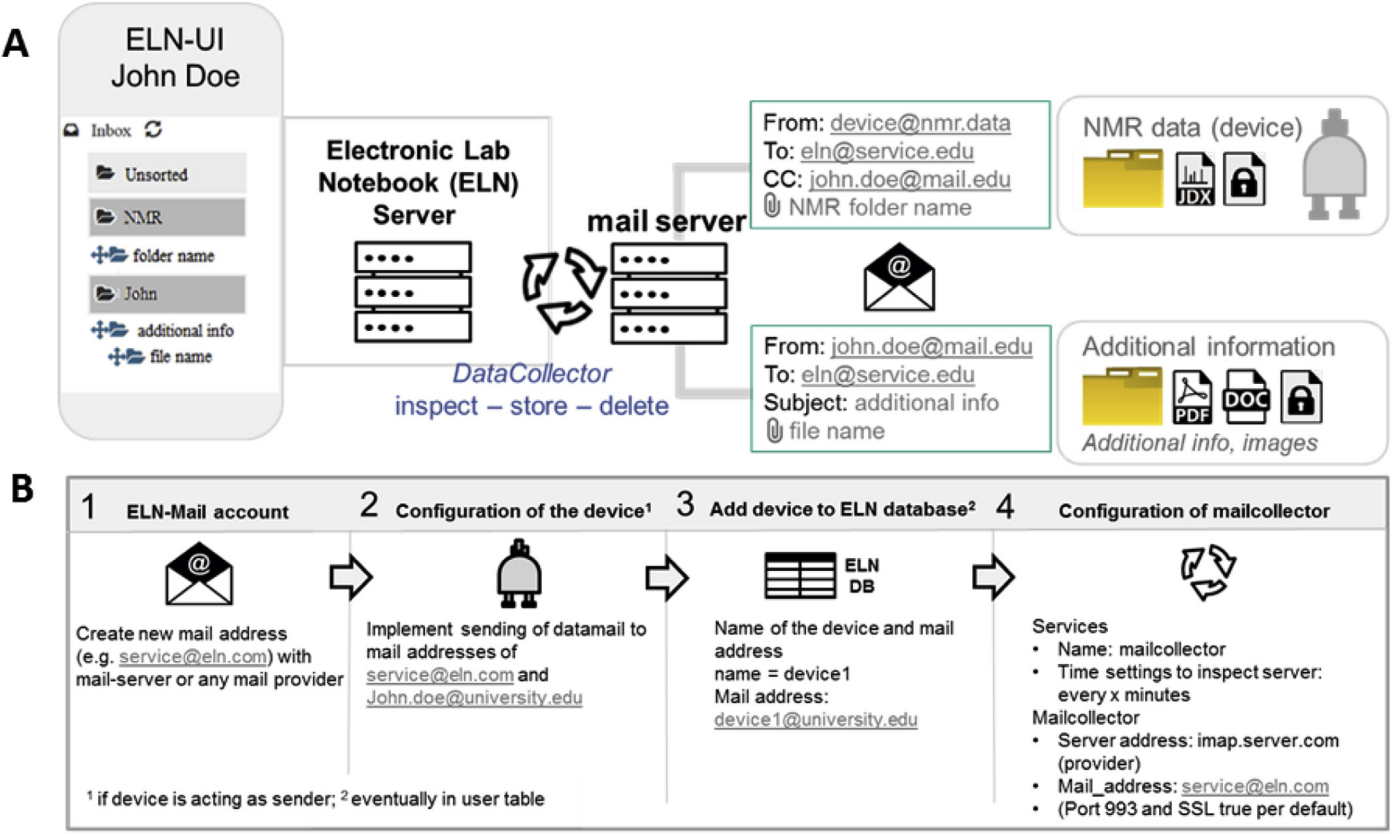
**A.** Data collection via the *MailCollector* routine implemented exemplarily for Bruker NMR spectrometers and additional information sent by email. **B.** Configuring the data transfer via the *MailCollector*.

One of the easiest possibilities to transfer data to a management system for research data (as the Chemotion-ELN) is to send data per email. The software of some analytical devices support the mailing of measurements to a user defined email address by default. This allows to implement storage routines that are independent of any additional software or hardware on the instrument's side. The only prerequisite for a use of the *MailCollector* routine is fulfilled if one can set the email to be send to two addressees, to the ELN server associated email address and to the user's email address registered with the corresponding ELN account (Fig. 2). This is necessary to identify the target of the mailed information and the corresponding account in the ELN. In short, four configuration steps are necessary to fetch the information via email:

• First, it is necessary to have an email account for the ELN with any provider or server that supports Internet Message Access Protocol (IMAP) to centrally collect the emailed data (step 1, Fig. 2B).
• Second, the device software has to be configured to send the data to the user's (scientist's) email account as a main recipient and to this ELN email address as a main recipient or in cc (step 2, Fig. 2B).
• Third, the devices sending emails should be whitelisted by the ELN server. For this, the devices information is persisted in the ELN DB as ‘device’ entities. The device class is a subclass of the ELN User class and each device's email can then be registered (step 3, Fig. 2B).
• The last step for the implementation of the mailing routine is the activation of the *MailCollector* service within the Chemotion ELN (this is also done by the administrator of the ELN server). For this, the credentials (email address and password) and email server IMAP settings for the ELN mail account as well as the cron time schedule parameters are configured (step 4, Fig. 2B).

The *MailCollector* (see Fig. 2A for the used routine) screens the ELN account for new emails and processes the data subsequently. The processing follows the routine: inspect – store– delete. New emails are inspected with respect to the sender (device) and the recipient (user). If both are known to the *MailCollector*, the service adds the data in the email's attachment to the ELN server and deletes the mail from the inbox of the ELN email account. All steps of the process can be logged. The routine is queued as a delayed_job task, executed at a specified time and requeued for execution according to the cron styled schedule setting. The described procedure is used in our group exemplarily for the transfer of data from Bruker NMR devices to the Chemotion ELN. The ELN server checks and processes its emails every 15 min as the standard routine to allow ELN users the access to their analytical data. The *MailCollector* code is given in the Supporting Information. Additionally, the mailing procedure is very helpful if the information to be added to an ELN is captured with a mobile device or is available on a computer. In this manner, almost all data, independent of its origin, can be transferred to the ELN. The only step necessary for this procedure is sending an email (using the same email address the user is registered with in the ELN) to the ELN's email account. Data that is attached to the email will be processed according to the *MailCollector* as described above and will be assigned to the user ELN account according to the email address of the sender (Fig. 2A).

### 2.3 *DataCollectors* for devices with LAN/WLAN (Procedure 2)

**Fig. 3:**
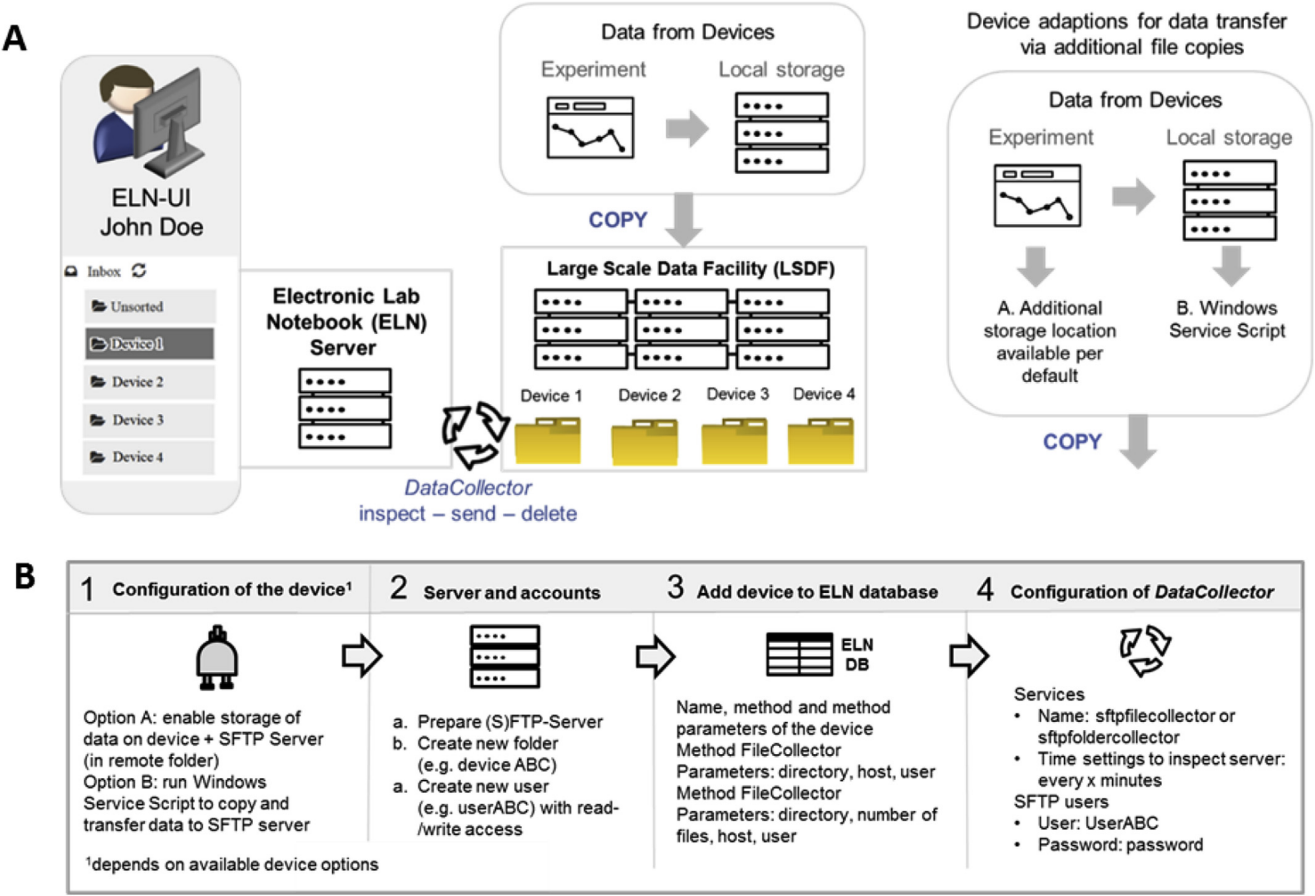
**A.** Left: Data collection via *DataCollectors* (*FileCollector* and *FolderCollector*) as implemented for the devices GC (MS), LC (MS), Raman and mass spectrometer (general labeling as Device 1 to Device 4). Right: Device configuration for data transfer via additional file copies as described in detail in the following chapter 4. **B.** Workflow of the installation of a data transfer routine via a *DataCollector*.

There are two possibilities to enable an automatic transfer of data to the ELN from devices that store data on a local computer or server. Either the data is actively transferred via a client application from the local computer/server to the ELN server or the ELN server directly accesses the device data storage folder. The first solution requires more efforts for the installation and maintenance of several client applications, therefore, we followed a central solution (Fig. 3A) where both, the device computer and the ELN server, have access to a common storage. Considering the availability of transfer software packages for the server and for the device computers, we focused on SFTP over NFS, CIFS/SAMBA, FTPS to securely transfer files. SFTP is relatively well supported and free third-party software can be found even for legacy operation systems (OS) such as windows XP. Though the latter OS supports mapping of the network drive, the obsolete and unsafe SMBv1 protocol cannot be accepted. Having such an OS in a network is nowadays a high security issue. These issues can be circumvented by entirely isolating the computer and device network and configuring a firewall exception for ssh communication with the external remote storage.

To allow the ELN to access the data, four basic adaptions are implemented:

• In a first step, the device has to be prepared to enable data storage on a remote folder (SFTP server) in addition to a data storage on a local computer or server. This configuration of the devices can be easily done if the instrument offers this option by default. In all other cases, a few changes have to be made according to the description given below in the subchapter “necessary device adaptions” (step 1, Fig. 3B).
• Second, folders for each device are created on a remote network drive. File access credentials are also generated. In our implementation, the central storage was provided by the Large Scale Data Facility, LSDF, in Karlsruhe, and a read/write account was created for each device folder. The results of the measurements are mirrored on this central storage in the folder reserved for the device. In addition, a copy of the raw data is being kept locally (step 2, Fig. 3B).
• In a third step, the device information along with a specific profile are registered to the database of the ELN as described in Procedure 1. The necessary information added to the new device entry in the DB includes the network drive (or server) for the remote folder, the corresponding path and the access credentials (step 3, Fig. 3B).
• The last step of the implantation consists of the activation of a *DataCollector* through the corresponding configuration file of the ELN server (step 4, Fig. 3B). Similar to the *MailCollector*, it requires a cron schedule type parameter at which the *DataCollector* becomes active. It also accepts the network credentials for sftp password authentication (instead of storing the passwords, it is also possible to configure rsa key authentication).

With the *DataCollector* activated, all data stored on remote accounts are processed at the set times. This routine works for all instruments that are connected to the collector and belong to the devices that are registered in the ELN. The *DataCollector* is a routine that works similar to the *MailCollector* described in Procedure 1, but uses, in contrast to the *MailCollector*, SFTP. Two different *DataCollector* types (*FileCollector* and *FolderCollector*) were developed and are used depending on whether the device stores measurement data either as a single file or as a folder including a defined number of objects. The *FileCollector* reads single data files which were written into the predefined remote folder, while the *FolderCollector* inspects the remote folder of a device for new subfolders (the codes of the *FileCollector* and *FolderCollector* are given in the Supporting Information). It is critical for this mode to know if the measurements have been completed and all files have been written. This can be achieved by defining the number of files that have to be present within a data folder for a completed measurement. If all data has been written, the expected file number is reached and the folder can be processed and assigned to an ELN user. All files are compressed in a ZIP folder and become accessible through the ELN account of the identified ELN user. To allow the assignment of the data collected by the *DataCollectors* to a specific ELN user, only a few rules have to be respected: the name of the file has to start with a unique identifier registered with the ELN user account. This identifier has to be separated from the experiment number via a hyphen or a period (e.g. JD-xyz for the registered user John Doe and a measurement xyz). If the initials can be assigned to a registered ELN user, the data is registered in the ELN account for the identified user. The processing of data by the *DataCollectors* follows the same routine “inspect – store – delete” as used in case of the *MailCollector*. All data that is found by the *DataCollectors* within the remote folders is transferred to the ELN server and deleted from the remote folder. Deleting successfully transferred data is of high importance to maintain a good performance of the ELN and data transfer server, as the amount and size of datasets increase with the running time and the resources required for their inspection increases as well. The deletion of the data in the remote folder is also done if no user of the ELN can be assigned to the datasets. All changes are documented in a log file.

#### 2.3.1 Necessary device adaptions

Using the *DataCollector* procedure requires the original data to be mirrored to a remote (network) folder. This depends on the device software itself and the operating system running it. This publication serves as a guide, exemplarily showing how to solve these challenges for common instruments (for selected suppliers). The solutions are demonstrated for Windows systems (see Fig. 3A, right part) and analytical measurements with devices such as GC, GCMS, HPLC, LCMS, MS, Raman and UV-VIS instruments. In those cases, where the device software does not support the storage of data at different places, it can be configured to transfer data via a Windows Service Script. A necessary requirement is that the device computer runs with an up to date (Windows) OS with PowerShell and NET Framework. The script (given in the Supporting Information) creates a copy of the measurement data to a remote folder on a network drive (in our case LSDF). For devices running on Windows 7 or newer operating systems, the script can be integrated as described above (and in further detail in the supporting Information), while devices running legacy operating systems (like Windows XP) need to be modified with additional software to allow secure data transfer protocols (please see Supporting Information chapter 3.3 for further details). The code (network paths and credentials given are placeholders) runs in Microsoft PowerShell and depends on. NET Framework. The script should be embedded as an. exe file and installed as a Windows Service application to allow a user-friendly implementation. [24] For a use on Linux operation system, the code can be adapted as a cron job with the general procedure working similarly.

### 2.4 *DataCollector* for devices without network connection (service devices) (procedure 3)

**Fig. 4:**
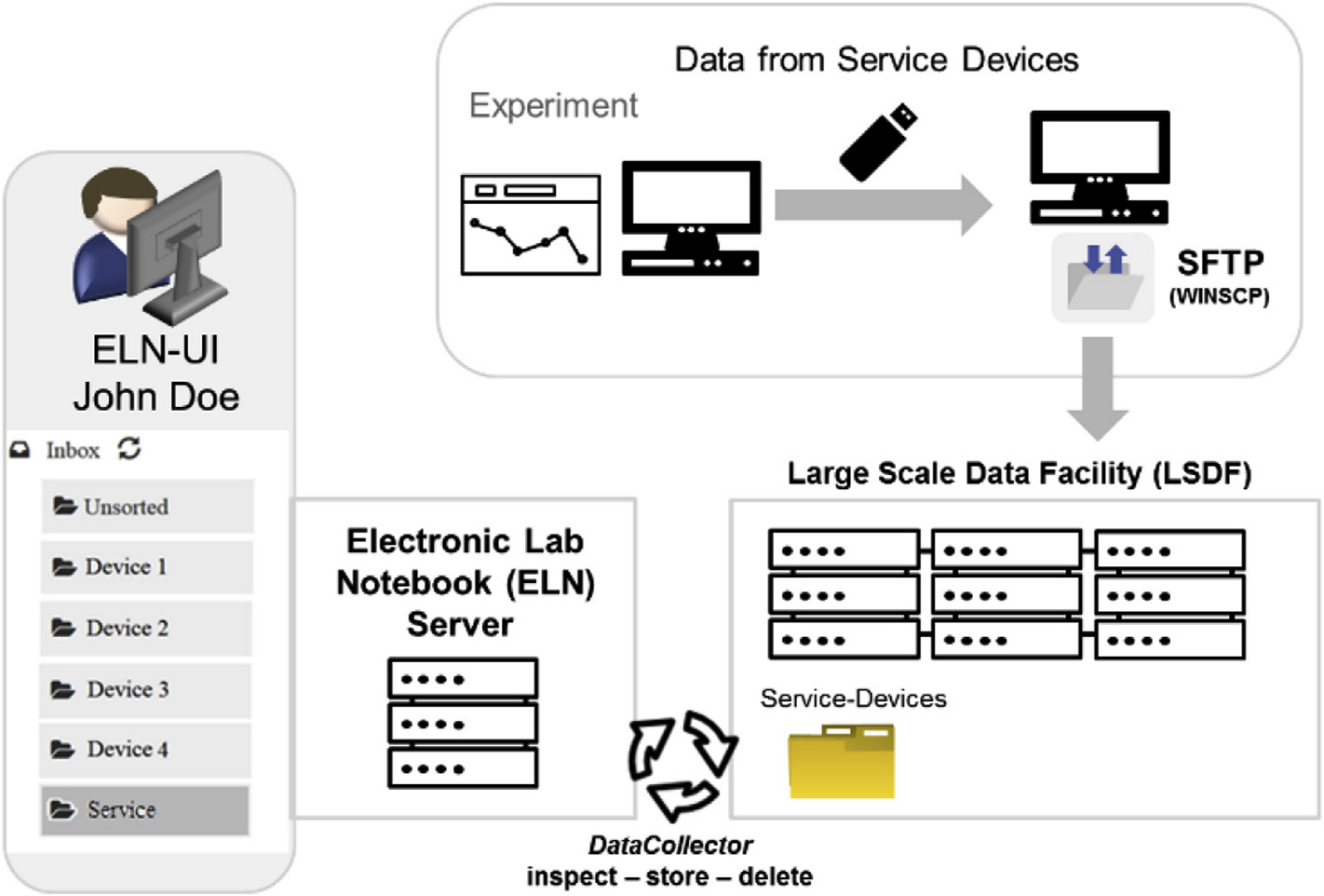
Procedure for data collection via *DataCollectors* applied to instruments without network connection (service devices).

The transfer of data from devices that have network access (either local network or internet access) can be achieved either via Procedure 1 or 2, while the transfer of data generated on devices that have no network connection is rather difficult. New instruments usually enable networking and support the newest transfer protocols, but many legacy devices lack this option. Additionally, some service departments in universities do not connect their instruments to any network to avoid undesired access or malware infection. For such cases, we designed a workflow that requires the manual transfer of measurement data from the device to a remote storage location with a portable storage device as an additional step (Fig. 4). All data is copied to the portable storage (e.g. a USB stick) and is then transferred to a remote folder from a computer with network connection. The procedure chosen in our approach was optimized via the SFTP protocol (via WinSCP). Another procedure that enables the successful transfer of data was tested via the access to a network drive which stores directly to the remote folder (LSDF, procedure not shown in Fig. 4). All data that is copied to the remote folder is processed according to Procedure 2 by the *FileCollector* as described before.

### 2.5 Adaptions to the ELN and design of the user interface

The developments described in the previous chapters are simple prerequisites that allow transferring data from several devices to a central storage (network drive) where it can be processed. Additionally, several adaptations to the Chemotion ELN backend as e.g. the implementation of the *DataCollectors* were also described in the previous sections. In a last step, adaptions to the User Interface of the ELN have been developed to allow a convenient management and visibility of the collected data. These changes are described in the following section. The Chemotion ELN was adapted by the addition of a management tool that is named *Inbox*. The *Inbox* collects all data, and allows the assignment of this data to a particular folder according to origin of the provided data file. All incoming files are indexed and the number of new data is given. Data transferred to the ELN by the *MailCollector* is either listed in a folder that is named according to the device that sent the data or according to the sender of the email (user of the ELN). The first routine runs if any device routine e.g. for NMR was implemented for the data transfer, the second option appears if additional data without reference to a registered device are added. For the latter routine, a subfolder lists all incoming mails according to the given subject of the email. For data collected by a *DataCollector*, the *Inbox* generates a folder for each device that is registered in the ELN and labels the folder according to the name of the device in the ELN DB. Empty folders can be deleted by the user if desired and are generated automatically again if new data from the corresponding device is collected. While the metadata e.g. the description of the experiment (which is given automatically with the transferred data) could in principle be used to assign data to its corresponding experiment in the ELN, we chose to use only the information ‘device type’ to sort data into the device folder (plus the date of the measurement for a chronological folder-based structuring of the data). The additional assignment of the data to an experiment is done manually by the user to avoid errors due to a mislabeling of measurements. The user can assign data to a selected sample (or reaction) analysis in the ELN by drag-and-drop of the data element (either single files or folders). The assignment to the experiment can be reversed, which results in the data element being returned to the original folder of the *Inbox.*

## 3 Conclusion

We developed a hands on-approach to solve challenges in analytical data management with focus on data capture and data storage. Data management in universities is a challenging endeavor in particular due to the diverse infrastructure of devices and software in combination with limited budget. To allow the storage and management of in particular the analytical measurements and data sets in a well-organized manner, we propose the use of an electronic lab notebook (ELN) in combination with small adaptions to commonly used analytical instruments and their data storage workflow. The given instruments were chosen according to the needs of synthetic chemists, in particular devices needed in organic, inorganic and polymer chemistry. The presented method is a low cost solution as all components that are necessary to install and use the herein elaborated functions, including the ELN which has been described earlier by our group, are available as Open Source. The source code can be deployed as server infrastructure. The established procedures include data transfer options from ubiquitous devices like NMR instruments, GC (MS) or LC (MS), IR and Raman, or mass spectrometers to a central server and the visualization of the available data files in the ELN. The designed workflows are described in a step by step procedure to allow the adaption of procedures in other universities accordingly if desired.

## Author contributions

The manuscript was written through contributions of all authors. All authors have given approval to the final version of the manuscript.

## Notes

The PowerShell script examples are also available via git at: https://git.scc.kit.edu/ComPlat/chemotion_eln_data_mirroring.

The source code of the Chemotion_ELN is available at: https://git.scc.kit.edu/ComPlat/chemotion_eln_server and https://github.com/ComPlat/chemotion_ELN.

Installation notes for Chemotion ELN server can be found at: https://git.scc.kit.edu/ComPlat/chemotion_eln_server/wikis/home.

A Virtual Machine (VM) template with preinstalled Chemotion ELN for production or development environments can be accessed at:

https://git.scc.kit.edu/ComPlat/chemotion_eln_server/wikis/vm-template.

## Declaration of interests

The authors declare that they have no known competing financial interests or personal relationships that could have appeared to influence the work reported in this paper.

## Abbreviations

LIMS: Laboratory and Information Management System
ELN: Electronic Laboratory Notebook
CSS: Cascading Style Sheets
DB: database
OS: operation system
UI: User Interface
LSDF: Large Scale Data Facility
SFTP: Secure File Transfer Protocol
SMB: Server Message Block
FTPS: File Transfer Protocol over SSL.

## Appendix A Supplementary data

The Supporting Information covers technical aspects and details of the software and programming e.g. the developed code fragments necessary to adapt the device computers for data transfer and the code used for the implementation of the *DataCollectors*. The Supporting Information additionally contains a hands-on-guide to step by step adaptions to the infrastructure to meet the requirements for the herein described data management options and advice for data transfer from legacy devices (secure protocols). Detailed descriptions of the configuration of the ELN DB and its influence on the UI of the ELN concerning the visibility of the measurements are given.
