# Supplementary material for: Procedures for systematic capture and management of analytical data in academia
Source: Anal Chim Acta X. 2019 Feb 15;1:100007. doi: 10.1016/j.acax.2019.100007 (PMC7587027; doi:10.1016/j.acax.2019.100007)
Supplement: Supplemental Material [file mmc1.pdf]

Procedures for Systematic Capture and Management of Analytical Data in Academia.

Jan Potthoff[a], Pierre Tremouilhac[b], Patrick Hodapp[b], Bernhard Neumair[a], Stefan Bräse*[b,c], Nicole Jung*[b,c]

Email: jan.potthoff@kit.edu; pierre.tremouilhac@kit.edu; patrick.hodapp@kit.edu; nicole.jung@kit.edu; neumair@kit.edu; braese@kit.edu

[a]Steinbuch Centre for Computing, Karlsruhe Institute of Technology, Hermann-von-Helmholtz-Platz 1, 76344 Eggenstein-Leopoldshafen, Germany; [b]Institute of Toxicology and Genetics, Karlsruhe Institute of Technology, Hermann-von-Helmholtz-Platz 1, 76344 Eggenstein-Leopoldshafen, Germany; [c]Institute of Organic Chemistry, Karlsruhe Institute of Technology, Fritz-Haber-Weg 6, 76131 Karlsruhe, Germany.

Contents

1. Implemented devices/brands and methods 3

2. Code for *DataCollectors* 3

2.1. Code *MailCollector* 3

2.2. Code *FileCollector* 4

2.3. Code *FolderCollector* 5

2.4. Code for Windows Service Script 8

3. Additional Information for the configuration of devices 8

3.1 Macro for the storage of Agilent GC/HPLC Data to two locations 8

3.2 Additional Information for the generation of a Windows Service Script 9

3.3. Additional Support for Devices running on old operation systems (Windows XP and older) 9

4. Additional Source Code availability 9

1. Implemented devices/brands and methods

Type	Manufacturer	Instruments	Ethernet	OS*	Method (Procedure)
NMR	Bruker	Avance 400 MHz	Yes	Linux	Mailcollector (1)
Mass spectrometer	Advion	Advion expression CMS	Yes	Win10	Config. B + FileCollector (2)
GC-MS	Agilent	GC: Agilent 6890<br>MS: Agilent 5973 inert	Yes	WinXP	Config. A + FolderCollector (2)
HPLC	Agilent	1100 Series	Yes	WinXP	Config. A + FolderCollector (2)
LCMS	Agilent	1100 Series	Yes	WinXP	Config. A + FolderCollector (2)
Raman spectrometer	Bruker	MultiRam	Yes	WinXP	Config. B + FileCollector (2)
IR spectrometer	Bruker	Alpha	No	WinXP	DataCollector (3)
UV-VIS spectrometer	Analytik Jena	Specord 50 plus	No	Win7	DataCollector (3)

*Operation system

2. Code for *DataCollectors*

2.1. Code *MailCollector*

```
def execute
	begin
		imap = Net::IMAP.new(@server, @port, @ssl)
		response = imap.login(@mail_address, @password)
		if response['name'] == 'OK'
			imap.select('INBOX')
			imap.search(['NOT', 'SEEN']).each do |message_id|
				process_new_mail(message_id, imap)
			end
			imap.close
		else
			log_error('Cannot login ' + @server)
			raise
		end
	ensure
		imap.logout
		imap.disconnect
	end
end

def process_new_mail(message_id, imap)
	begin
		raw_message = imap.fetch(message_id,
		'RFC822').first.attr['RFC822']
		message = Mail.read_from_string raw_message
		helper = create_helper(message)
		log_info 'Mail from ' + message.from.to_s
```

```
			unless helper
				log_info message.from.to_s + ' Email format incorrect!'
				return nil
			end
			unless helper.sender
				log_info message.from.to_s + ' Sender unknown!'
				return nil
			end
			unless helper.recipient
				log_info message.from.to_s + ' Recipient unknown!'
				return nil
			end
			if message.attachments
				store_new_data(message, helper)
				log_info message.from.to_s + ' Data stored!'
			else
				log_info message.from.to_s + ' No data!'
			end
			imap.store(message_id, '+FLAGS', [:Deleted])
			log_info message.from.to_s + ' Email processed!'
		rescue => e
			log_error e.backtrace.join('\n')
		end
end
```

2.2. Code *FileCollector*

```
def execute(use_sftp)
		devices(use_sftp).each do |device|
		if use_sftp
			credentials = Rails.configuration.datacollectors.sftpusers.select { |e|
      e[:user] == device.profile.data['method_params']['user']
    }.first
    if credentials
      Net::SFTP.start(
        device.profile.data['method_params']['host'],
        credentials[:user],
        password: credentials[:password]
      ) do |sftp|
        @sftp = sftp
        inspect_folder(device)
      end
    end
   else
    @sftp = nil
    inspect_folder(device)
   end
  end
 end

 private
 def devices(use_sftp)
  use_sftp ? search_for = 'filewatchersftp' : search_for = 'filewatcherlocal'
  Device.all.select { |e|
   e.profile.data && e.profile.data['method'] == search_for
  }
 end

 def inspect_folder(device)
```

```
    directory = device.profile.data['method_params']['dir']
    new_files(directory).each do |new_file_p|
      @current_file = DatacollectorFile.new(new_file_p, @sftp)
      error = CollectorError.find_by error_code: CollectorHelper.hash(
        @current_file.path,
        @sftp
      )
      begin
        stored = false
        if @current_file.recipient
          unless error
            @current_file.collect_from(device)
            log_info 'Stored!'
            stored = true
          end
          @current_file.delete
          log_info 'Status 200'
        else # Recipient unknown
          @current_file.delete
          log_info 'Recipient unknown. File deleted!'
        end
      rescue => e
        if stored
          CollectorHelper.write_error(
            CollectorHelper.hash(@current_file.path, @sftp)
          )
        end
        log_error e.backtrace.join('\n')
      end
    end
  end

  def new_files(monitored_folder_p)
    if @sftp
      new_files_p = @sftp.dir.glob(monitored_folder_p, '*').reject(
        &:directory?
      )
      new_files_p.map! do |f|
        File.join(monitored_folder_p, f.name)
      end
    else
      new_files_p = Dir.glob(File.join(monitored_folder_p, '*')).reject { |e|
        File.directory?(e)
      }
    end
    new_files_p.delete_if do |f|
      f.end_with?('.filepart', '.part')
    end
    new_files_p
  end
```

2.3. Code *FolderCollector*

```
def execute(use_sftp)
  unless Rails.configuration.datacollectors
    raise 'No datacollector configuration!'
  end
  devices(use_sftp).each do |device|
```

```
    if use_sftp
      credentials = Rails.configuration.datacollectors.sftpusers.select { |e|
        e[:user] == device.profile.data['method_params']['user']
      }.first
      if credentials
        Net::SFTP.start(
          device.profile.data['method_params']['host'],
          credentials[:user],
          password: credentials[:password]
        ) do |sftp|
          @sftp = sftp
          inspect_folder(device)
        end
      end
    else
      @sftp = nil
      inspect_folder(device)
    end
  end
end

private

def devices(use_sftp)
  if use_sftp
    Device.all.select { |e|
      e.profile.data && e.profile.data['method'] == 'folderwatchersftp'
    }
  else
    Device.all.select { |e|
      e.profile.data && e.profile.data['method'] == 'folderwatcherlocal'
    }
  end
end

def inspect_folder(device)
  params = device.profile.data['method_params']
  new_folders(params['dir']).each do |new_folder_p|
    @current_folder = DatacollectorFolder.new(new_folder_p, @sftp)
    @current_folder.files = list_files
    error = CollectorError.find_by error_code: CollectorHelper.hash(
      @current_folder.path,
      @sftp
    )
    begin
      stored = false
      if @current_folder.recipient
        if @current_folder.files.length != params['number_of_files']
          log_info 'Wrong number of files!'
          next
        end
        unless error
          @current_folder.collect(device)
          log_info 'Stored!'
          stored = true
        end
        @current_folder.delete
        log_info 'Status 200'
```

```
      else # Recipient unknown
        @current_folder.delete
        log_info 'Recipient unknown. Folder deleted!'
      end
    rescue => e
      if stored
        CollectorHelper.write_error(
          CollectorHelper.hash(@current_folder.path, @sftp)
        )
      end
      log_error e.backtrace.join('\n')
    end
  end
end

def list_files
  if @sftp
    all_files = @sftp.dir.entries(@current_folder.path).reject(
      &:directory?
    )
    all_files.map!(&:name)
  else
    all_files = Dir.entries(@current_folder.path).reject { |e|
      File.directory?(File.join(@current_folder.path, e))
    }
  end
  all_files.delete_if do |f|
    f.end_with?('..', '.', '.filepart', '.part')
  end
  all_files
end

def new_folders(monitored_folder_p)
  if @sftp
    new_folders_p = @sftp.dir.glob(monitored_folder_p, '*').select(
      &:directory?
    )
    new_folders_p.map! { |dir| File.join(monitored_folder_p, dir.name) }
  else
    new_folders_p = Dir.glob(File.join(monitored_folder_p, '*')).select { |e|
      File.directory?(e)
    }
  end
  new_folders_p
end
```

2.4. Code for Windows Service Script

```
# Configure local and remote folders
$folder = 'D:\Advion Mass Express\1.0\Data'
$filter = '*.datx'
$destination = '\\server\Advion_MS\data'
$destination2 = '\\server\Advion_MS\ELN'

# Configure network credentials and shared network location
$username = 'user'
$password = 'passw0rd'
net use '\\server\Advion_MS\' $password /USER:$username

#The following monitors for events and triggers an action
$fsw = New-Object IO.FileSystemWatcher $folder, $filter -Property @{
IncludeSubdirectories = $true
NotifyFilter = [IO.NotifyFilters]'FileName, LastWrite'
}
$onCreated = Register-ObjectEvent $fsw Created -SourceIdentifier FileCreated -Action {
$path = $Event.SourceEventArgs.FullPath
start-sleep -s 5
Copy-Item $path -Destination $destination -Force
Copy-Item $path -Destination $destination2 -Force
}
```

3. Additional Information for the configuration of devices

3.1 Macro for the storage of Agilent GC/HPLC Data to two locations

```
name ELN_Export
        Filesize "Z:\connection.txt"
        If size > -1 then
                COPY _DataPath$ + _DataFile$, "Z:\ELN\" + _DataFile$, DONTASK
                COPY _DataPath$ + _DataFile$, "Z:\data\" + _DataFile$, DONTASK
        Else
                Alert "No connection to the LSDF; Your data could not be backed up and exported to the
ELN.",2
        Endif
remove ELN_Export
```

Export of measurement data to a network location for systems not supporting simultaneous storage of measurement data to two locations was done by adding a macro to the method to be run after

every measurement. An exemplary macro for Agilent devices running ChemStation is shown above. The method must be edited to run the macro after every completed measurement and the mapped network drive must include a test file (here connection.txt). The script consists of two parts: first the connection to the network drive is tested by checking that the file “connection.txt” can be accessed and the system has read/write privileges to the remote location. If the connection test is successful the measurement data will be copied to the remote location; if the test file cannot be accessed a message box will appear alerting the user that there was a connection problem and the data could not be copied.

3.2 Additional Information for the generation of a Windows Service Script

For the transfer of data to the folder monitored by the *FileCollector* or *FolderCollector* the remote folder can be mapped as a network drive. Once the network drive is mapped it can be set as a backup location or second storage location so all data is stored locally in addition to the remote folder. For software not supporting simultaneous saving of data to two locations, a macro or script can be amended to the method and run after a completed measurement to copy the data to the remote folder (as presented in section 3.1). For Software not supporting either one of those two options an additional script, like the one in section 2.4 can be used to copy the data to the remote storage location.

Although it is possible to run the script from method 2.4 in PowerShell after booting the PC, the PowerShell window has to stay open and active constantly for the data to be copied. It is therefore possible to convert the script to an .exe file and run it as a windows service. After booting Windows the service is started automatically and runs in the background. (There are several options to convert the script to an executable file and set that up as a Windows Service Script, the recommended one refers to a well-documented protocol: https://pastebin.com/5r1XfftG)

3.3. Additional Support for Devices running on old operation systems (Windows XP and older)

Due to the lack of support from Microsoft for outdated operating systems such as Windows XP and because in many cases the licensed software for old analytical devices is restricted to an old operating system, solutions for data transfer from these computers to the remote folder monitored by the *FolderCollector* and *FileCollector* may be an obstacle. Modern data centers will usually not allow connection requests with old data transfer protocols (such as SMBv1), making it impossible to map network drives. As a workaround programs that allow mapping of network drives using the SFTP protocol can be used (such as SFTP Net Drive http://www.sftpnetdrive.com).

4. Additional Source Code availability

The PowerShell script examples are also available via git at:
https://git.scc.kit.edu/ComPlat/chemotion_eln_data_mirroring

The source code of the Chemotion_ELN is available at:
https://git.scc.kit.edu/ComPlat/chemotion_eln_server and
https://github.com/ComPlat/chemotion_ELN

Installation notes for Chemotion ELN server can be found at:
https://git.scc.kit.edu/ComPlat/chemotion_eln_server/wikis/home

A Virtual Machine (VM) template with preinstalled Chemotion ELN for production or development environments can be found at:
https://git.scc.kit.edu/ComPlat/chemotion_eln_server/wikis/vm-template
